# Lentiviral-Mediated Silencing of Farnesyl Pyrophosphate Synthase through RNA Interference in Mice

**DOI:** 10.1155/2015/914026

**Published:** 2015-01-22

**Authors:** Jian Yang, Chen-Ze Zhao, Bin Chen, Fei Chen, Jie Han, Shen-Jiang Hu

**Affiliations:** Institute of Cardiology, The First Affiliated Hospital, College of Medicine, Zhejiang University, Hangzhou 310003, China

## Abstract

Farnesyl pyrophosphate synthase (FPPS) plays a vital role in the mevalonate pathway and has been shown to be involved in hypertrophy and cardiovascular diseases. Lentivirus-mediated RNA interference (RNAi) to knock down a gene of interest has become a promising new tool for the establishment of transgenic animals. The interfering fragment, named pLVT202, was chosen from cardiomyocytes tested *in vitro* and was microinjected into the perivitelline space of zygotes from C57BL/6J mice via a lentivirus vehicle; 20 were identified as carrying copies of the transgene using the polymerase chain reaction (PCR). Real-time PCR and western blotting analysis showed that FPPS was downregulated in multiple tissues in the transgenic mice. The transgenic mouse model provides a novel means of studying the gene function of FPPS.

## 1. Introduction

To date, it has been shown that farnesyl pyrophosphate synthase (FPPS) plays a vital role in the mevalonate pathway, which is essential for forming cholesterol and isoprenoids. In a key step, FPPS catalyzes the formation of geranyl pyrophosphate (GPP) and farnesyl pyrophosphate (FPP). FPP is an important substrate not only in cholesterol and coenzyme Q biosynthesis, but also in the isoprenylation of small GTPases, such as Ras and Rho. It has been reported that FPPS expression is apparently upregulated in a number of diseases, including solid tumors [1], neurodegenerative diseases [2], and bone diseases [3]. Additionally, our previous studies suggest that the expression level of FPPS is significantly increased in angiotensin (Ang) II-treated cardiomyocytes as well as in hypertrophic heart tissues from spontaneously hypertensive rats (SHRs) [4, 5], while inhibition of FPPS by an inhibitor is beneficial in improving heart disease. However, the studies described here had limitations. SHRs are not the perfect model to examine the function of a specific gene because of their complex genetic background. Also, we could not exclude the other pharmacological effects of FPPS inhibitors, which could influence cardiac remodeling. Thus, it would be useful to establish a transgenic animal model for further study. Recently, we found that the cardiac-specific overexpression of farnesyl pyrophosphate synthase in mice induces cardiac hypertrophy and dysfunction [6], which highlighted the importance of FPPS in cardiovascular remodeling. However, no reports of FPPS knock-out or knock-down mice have been published to date, and whether downregulated FPPS expression leads to alleviation of ventricular remodeling in transgenic or knock-out mice remains unknown. Therefore, to facilitate functional studies of FPPS, we decided to establish a transgenic mouse in which the FPPS gene was silenced.

RNAi is a powerful tool for the analysis of gene function in various fields. It is triggered by double-stranded RNA (dsRNA) precursors that vary in length and origin. Lentiviruses are an attractive vehicle for gene transfer, integrating into the genome of nondividing cells. Furthermore, lentivirus-mediated transgenesis has become a basic approach because of the ability to infect embryonic stem cells and preimplantation embryos [7, 8]. Increasing evidence has shown that lentivirus-mediated delivery can express integrated small interfering RNA (siRNA) efficiently in a wide variety of cell lines, both* in vitro* and* in vivo*, and hundreds of transgenic models have been made by this technique [8, 9]. Previously, we showed that knock-down of FPPS expression with siRNA prevents Ang II-induced hypertrophy in cultured cardiomyocytes [4], and now we report the establishment of a FPPS gene silencing mouse model using lentiviral vectors expressing siRNA. These studies provide a new model for the study of FPPS function during cardiovascular remodeling and in other diseases* in vivo*.

## 2. Materials and Methods

### 2.1. Construction of siRNA and Lentiviral Packaging

**Figure 1:**
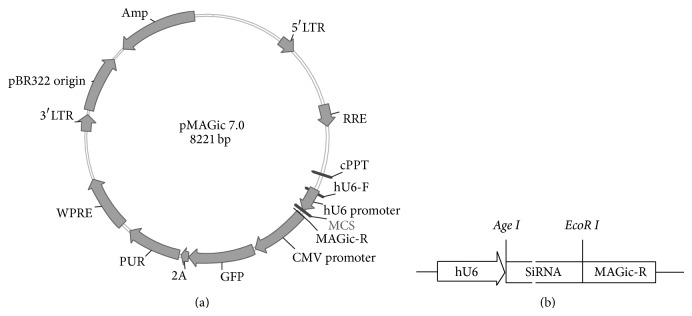
(a) Structure of lentiviral vector plasmid pMAGic 7.0. (b) Structure of pLVT vectors. The siRNA fragment was inserted using* Age *I and* EcoR *I.

Five individual sequences were designed to interfere with the murine FPPS gene and one nontargeting sequence was used as a control (Table 1). The double-stranded oligonucleotide was linked to a linear lentiviral vector plasmid pMAGic 7.0 (Sunbio, Shanghai, China) using* Age* I and* EcoR* I to produce short hairpin RNA (shRNA) vectors, termed pLVT202, pLVT274, pLVT275, pLVT276, pLVT277, and pLVT7 (Table 1, Figure 1), and* E. coli* strain DH5a was used as the plasmid host. The bacterium was cultured in Luria broth** (**LB) agar plates with appropriate antibiotic. The construct was confirmed by polymerase chain reaction (PCR) and DNA sequence analysis. Then, three-plasmid transient transfections of 293T cells were performed for lentivirus production, as previously reported [10].

### 2.2. Infection of Mouse Cardiomyocytes

Neonatal cardiomyocytes were prepared from the ventricles of 1-2-day-old C57BL/6J mice, obtained from the Experimental Animal Center, Chinese Academy of Sciences (Shanghai, China). The cardiomyocytes were cultured in medium with 10% FBS at 37°C for 24 h before initiating the lentivirus transfection experiment.

Cardiomyocytes were infected at a multiplicity of infection (MOI) of 50 pfu/cell in the culture medium for 72 h at 37°C in a humidified incubator with 5% CO_2_.

### 2.3. Animals

Mice were bred and housed under a 12/12 h light/dark cycle with free access to food and water at the Laboratory Animal Centre of Zhejiang University. The investigation conformed to the Guide for the Care and Use of Laboratory Animals, published by the US National Institutes of Health (NIH Publication Number 85-23, revised 1996), and was approved by the Institutional Animal Care and Use Committee of Zhejiang University.

### 2.4. Establishment of FPPS Interfering Transgenic Mice

The lentivirus was microinjected through the zona pellucida into the perivitelline space of zygotes of C57BL/6J mice. The injected zygotes were implanted into the oviduct of pseudopregnant foster mothers. The mice were impregnated and gave birth after the operation. Selected mice were mated with wild-type animals to keep their offspring at heterozygosity.

### 2.5. Identification of Transgenic Mice

Transgenic mice were identified by PCR analysis using tail DNA (forward primer 5′-GCAAATGGGCGGTAGGCGTGTA-3′, reverse primer 5′-TCGGGCATGGCGGACTTGAA-3′). PCR conditions were denaturation at 95°C for 5 min, followed by 40 cycles of 95°C for 30 s, 54°C for 30 s, and 72°C for 30 s, with a final extension at 72°C for 2 min. The PCR products were electrophoresed on a 1.2% agarose gel (GENE Company, Hong Kong, China).

### 2.6. Expression of FPPS Assessed by Real-Time PCR and Western Blotting

Total RNA was isolated from homogenized ventricular myocardium using an RNAiso kit (TaKaRa Bio, Tokyo, Japan). After digestion of the RNA with DNase I (TaKaRa Bio), first-strand cDNA was synthesized by reverse transcription (TaKaRa Bio). PCR was carried out with SYBR Premix Ex Taq (TaKaRa Bio) using 5 *μ*L of cDNA (which corresponded to 20 ng of total RNA in a final volume of 20 *μ*L) and 0.4 *μ*mol/L of each primer (Table 2). Quantitative PCR was carried out using ABI 7500 (Applied Biosystems, Foster City, CA, USA). Amplification specificity was checked using a melting curve following the manufacturer's instructions. Data were normalized according to the abundance of *β*-actin mRNA and then expressed relative to the mean value for the NTg mice group.

Prepared protein samples (30–50 mg) from several tissues from 6-week-old transgenic and control mice were separated by 12% sodium dodecyl sulfate-polyacrylamide gel electrophoresis (SDS-PAGE) and transferred onto polyvinylidene fluoride (PVDF) membranes. The membranes were blocked with 5% skim milk in Tween/TBS (TBST) for 1 h and subsequently incubated overnight at 4°C with rabbit polyclonal anti-FPPS antibody (ProteinTech) (1 : 1500 dilution) or mouse monoclonal anti-*β*-actin antibody (GenScript) (1 : 2,000 dilution). Membranes were washed three times with TBST and incubated with the secondary antibody (horseradish peroxidase-conjugated) at a dilution of 1 : 5000 at room temperature for 1 h. After washing four or five times with TBST, proteins were visualized with an enhanced chemiluminescence (ECL) detection reagent (Beit Haemek, Kibbutz Beit Haemek, Israel).

### 2.7. Histological Examination

Various tissues were dissected from 10-week-old mice and fixed in 10% (v/v) formalin. Sections were made from paraffin-embedded tissue samples, stained with hematoxylin and eosin, examined under a microscope, and photographed. Pathological analysis was carried out in the Department of Pathology, Zhejiang University, China.

## 3. Results

### 3.1. Identity of Constructs

The identities of the pLVT vectors were confirmed by PCR and DNA sequence analysis (data not shown), showing that all sequences of the interfering DNA were correctly inserted and linked.

### 3.2. Expression of FPPS Gene in Mouse Cardiomyocytes

**Figure 2:**
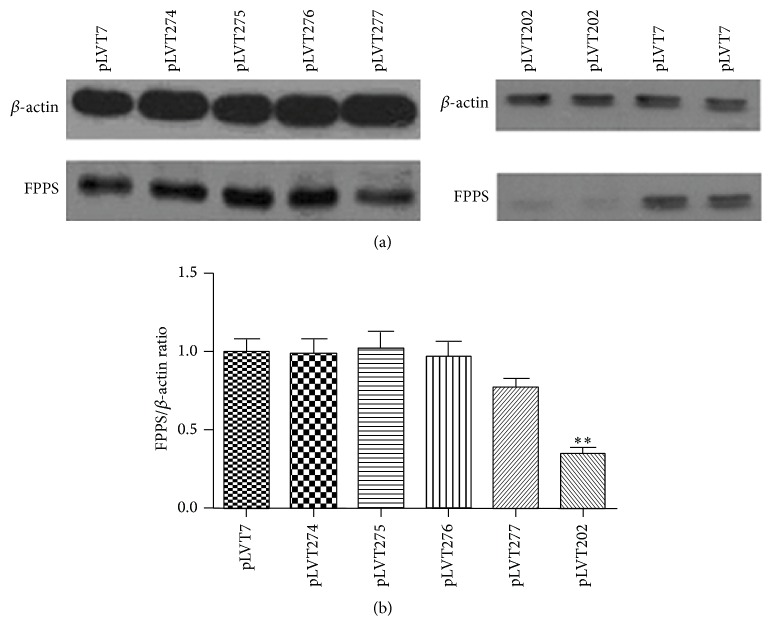
(a) Western blot data of FPPS expression in cardiomyocytes infected with different lentivirus. (b) Relative expression levels of FPPS normalized to *β*-actin are expressed as the mean ± SEM. Identical results were obtained in three independent experiments. ^**^ *P* < 0.01 versus pLVT7 (nontargeting control).

To evaluate the knock-down efficacy of shRNA, western blot analysis was performed. There was a clear decrease in the expression of the FPPS in the lentiviral infection group of pLVT202 compared with the control (about 65% loss), whereas the shRNA pLVT274-277 showed less knock-down efficacy than pLVT202 (Figure 2). Thus, it seemed that pLVT202 was the best choice.

### 3.3. Establishment of FPPS Interfering Transgenic Mice

**Figure 3:**
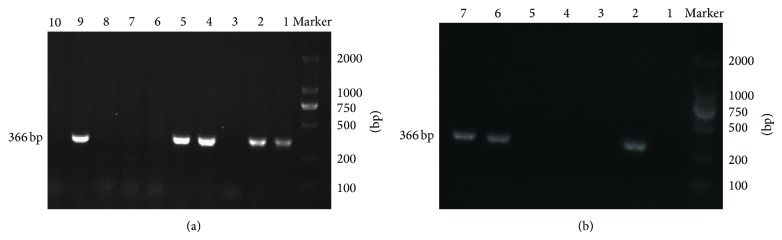
PCR analysis of transgenic mice. (a) Lanes 1, 2, 4, and 5: F1 transgenic mice; lanes 3 and 6–8: nontransgenic mice; lane 9: positive control (pLVT202); lane 10: negative control (wild-type mouse). (b) Lane 1: negative control (wild-type mouse). Lanes 2 and 6-7: F4 transgenic mice; lanes 3–5: nontransgenic mice.

The lentivirus containing the shRNA pLVT202 was microinjected through the zona pellucida into the perivitelline space of 375 fertilized oocytes of C57BL/6J mice. The injected zygotes were implanted into the oviducts of 15 pseudopregnant foster mothers, of which nine became pregnant and gave birth to 37 pups. By PCR analysis, 20 of them showed provirus integration. The ratio of transgenic integration was 54%. There was no apparent difference between the heterozygous mice and the wild-type in gross appearance. The transgenic mice bred normally with the wild-type to give birth to offspring, some of which carried the integrated fragment (Figure 3(a)).

### 3.4. FPPS Expression in Multiple Tissues of Transgenic Mice

**Figure 4:**
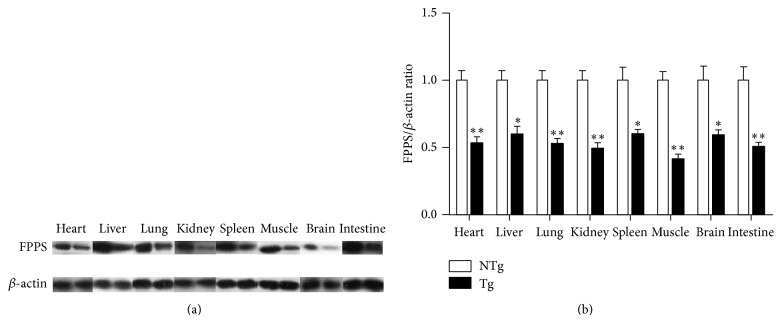
(a) Western blot data of FPPS expression in transgenic mice. Left: samples of each tissue were from nontransgenic mice and those on the right were from transgenic mice. (b) Relative expression levels of FPPS normalized to *β*-actin are expressed as the mean ± SEM (*n* = 3). NTg: non-transgenic; Tg: transgenic (F2). ^*^ *P* < 0.05; ^**^ *P* < 0.01 versus NTg mice.

**Figure 5:**
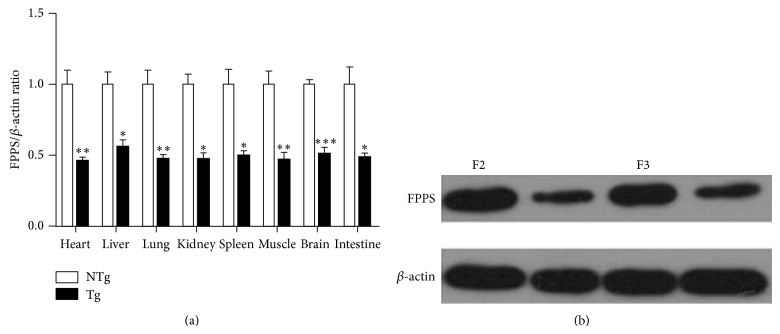
FPPS expression in transgenic mice. (a) Relative mRNA expression levels of FPPS normalized to *β*-actin are expressed as the mean ± SEM (*n* = 3). NTg: nontransgenic; Tg: transgenic (F2). ^*^ *P* < 0.05; ^**^ *P* < 0.01; ^***^ *P* < 0.001 versus NTg mice. (b) FPPS protein expression in F2 and F3 mice. Left: samples of heart tissue were from nontransgenic mice and those on the right were from transgenic F2 and F3 mice.

We analyzed FPPS expression in transgenic mice by real-time PCR and western blot analyses. FPPS mRNA and protein expression levels were efficiently downregulated in heart, liver, lung, spleen, kidney, brain, intestine, and skeletal muscle (Figures 4 and 5(a)). These results were consistent with previous studies [11, 12] that transgene expression in transgenic animals may differ between tissues.

### 3.5. The Stable Transmission and Continuous FPPS Suppression by RNAi in Mice

We used PCR to detect transmission of the shRNA allele in offspring mice (Figure 3(b)). FPPS protein expression levels were efficiently downregulated in the heart tissues of mouse offspring, as determined by western blot analysis (Figure 5(b)).

### 3.6. Pathological Analysis of Tissues

Histological analysis was carried out to determine whether the expression of the FPPS transgene caused any pathological changes. We found no obvious pathological changes in the tissues examined (data not shown). At least in the tissues that we examined, knockdown of FPPS did not cause any pathological consequences in the transgenic mice.

## 4. Discussion

To establish a transgenic RNAi model for the functional study of FPPS* in vivo*, we have, for the first time, generated transgenic mice using a lentiviral vector expressing siRNA targeting FPPS. Our previous studies showed that FPPS inhibition is beneficial in improving heart disease, such as hypertrophy [13–15] and endothelial function [16], while drugs, including nitrogen-containing bisphosphonates (NBPs), were the only way to suppress its expression before RNA interference [4]. NBPs are used primarily in the treatment of bone diseases [17]. Alendronate, one type of NBP, has also been used in cardiomyocyte research [13, 15]. Additionally, Wang et al. compared the effects of siRNA to alendronate and drew the conclusion that FPPS siRNA could promote preosteoblast cell differentiation without cytotoxicity while 50 *μ*M alendronate exhibited potent inhibition of cell viability [3]. This suggests that RNAi may be a safe and effective way to inhibit FPPS instead of small-molecule drugs. To the best of our knowledge, this is the first report on the knock-down of FPPS RNAi* in vivo*.

Lentiviral vectors that contain RNAi expression sequences could serve as a fast and effective alternative for the generation of mice with reduced expression of specific genes to the traditional knock-out strategy, which may lead to early embryo lethality, because FPPS is highly expressed during development [18]. The transgene introduced by lentiviral vectors is expressed during differentiation, with a lack of gene silencing in mammalian embryonic stem cells and preimplantation embryos, and represents a significant improvement over oncoretroviral vectors [7]. Additionally, the use of lentiviral vectors for transgenesis, combined with siRNA to silence gene expression, allows the microinjection of the transgenic fragment into the perivitelline space. These advantages can minimize the damage to zygotes and embryos and enhance the rate of survivors, compared with the traditional approach of microinjecting into the male pronucleus. In the present study, the ratio of transgenic integration was as high as 54%, and the interfering fragment expression in multiple F1 mice was shown to be effective. It has been shown that transgene expression levels decrease with aging* in vivo *from transgenic pigs [12]. However, there are few studies that cover stability in generating RNAi mice. In this study, we demonstrated that the integrated fragment could be detected by PCR in the F4 mice, and also continuous downregulation of FPPS was detected in the heart of F2 and F3 mice, which indicated the stable transmission of the lentivirus-mediated silencing cassette in our model.

In the present study, we described the establishment of a FPPS downregulation transgenic mouse model via lentivirus-mediated siRNA delivery. We demonstrated that FPPS downregulation was widespread, in multiple tissues of the transgenic mice. Interestingly, the expression of FPPS we observed in myocardium* in vivo* (53%) dropped less than that* in vitro* (35%), while lack of FPPS may trigger a positive feedback response in the mevalonate pathway* in vivo*. This may be a result of the upregulation of a large number of genes, other than the target gene. Moreover, although FPPS is involved in many diseases, no pathological changes occurred in the 10-week-old transgenic mice with FPPS knock-down, other than negative feedback, tumors, and neurodegenerative diseases, and cardiovascular diseases involve many factors and any pathological phenotype might require a long time to appear. Furthermore, FPPS RNAi mice might appear to be normal (i.e., like wild-type mice) until challenged with some physiological or pathological trigger.

In summary, we successfully developed a FPPS knock-down mouse model using RNAi. Once the effects of FPPS knockdown are confirmed* in vivo*, siRNA may even have potential clinical therapeutic implications. Along with a previous transgenic mouse model with FPPS overexpression we reported previously [11], the transgenic mouse model here provides a new means of studying FPPS gene function* in vivo*.

## 5. Conclusions

From this present study, we can conclude the following.

- The lentiviral shRNA of pLVT202 was the best choice for downregulating the FPPS gene* in vitro*, which reduced its expression by ~65%.
- We observed that 20 offspring mice showed provirus integration by PCR analysis. The frequency of transgenic integration was 54%.
- We successfully developed a FPPS knock-down mouse model using RNAi. FPPS downregulation was widespread in multiple tissues of the transgenic mice.
- The transmission of the lentivirus-mediated silencing cassette was stable and continuous suppression by RNAi was observed in our mouse model.

## Supplementary Material

In order to determine whether the expression of the FPPS transgene caused any pathological changes, we made pathological analysis on various tissues including intestine, liver, heart, lung, spleen, muscle, kidney and brain. As shown in Supplementary figure 1, there were no obvious pathological changes in the tissues examined.

## Figures and Tables

**Table 1:** Sequences of five siRNAs targeting murine FPPS and one nontargeting control.

Vector	Gene	Target sequence	GC%
pLVT202	FPPS	GACAGCTTTCTACTCTTTC	42.1
pLVT274	FPPS	CGCCAGATCTTAGAGGAGAAT	47.6
pLVT275	FPPS	GCTTTCTTCAAGTATGAGGAA	38.1
pLVT276	FPPS	GCCATGTGGATCTTGGTAGAT	47.6
pLVT277	FPPS	GCTTTCTTCCTTGTGTCAGAT	42.9
pLVT7	NC	TTCTCCGAACGTGTCACGT	52.6

NC: nontargeting control.

**Table 2:** Sequences of primers used for real-time polymerase chain reaction.

Name	Sequence	Product (bp)
Mouse-*β*-actin	F: 5′-TCATCACTATTGGCAACGAGC-3′	399
	R: 5′-AACAGTCCGCCTAGAAGCAC-3′	
Mouse-FPPS	F: 5′-GGAGGTCCTAGAGTACAATGCC-3′	155
	R: 5′-AAGCCTGGAGCAGTTCTACAC-3′	

F: forward; R: reverse.
