# Supplementary material for: Lentiviral-Mediated Silencing of Farnesyl Pyrophosphate Synthase through RNA Interference in Mice
Source: Biomed Res Int. 2015 Jan 22;2015:914026. doi: 10.1155/2015/914026 (PMC4320928; doi:10.1155/2015/914026)
Supplement: Supplementary file 1 — In order to determine whether the expression of the FPPS transgene caused any pathological changes, we made pathological analysis on various tissues including intestine, liver, heart, lung, spleen, muscle, kidney and brain. As shown in Supplementary figure 1, there were no obvious pathological changes in the tissues examined. [file 914026.f1.pdf]

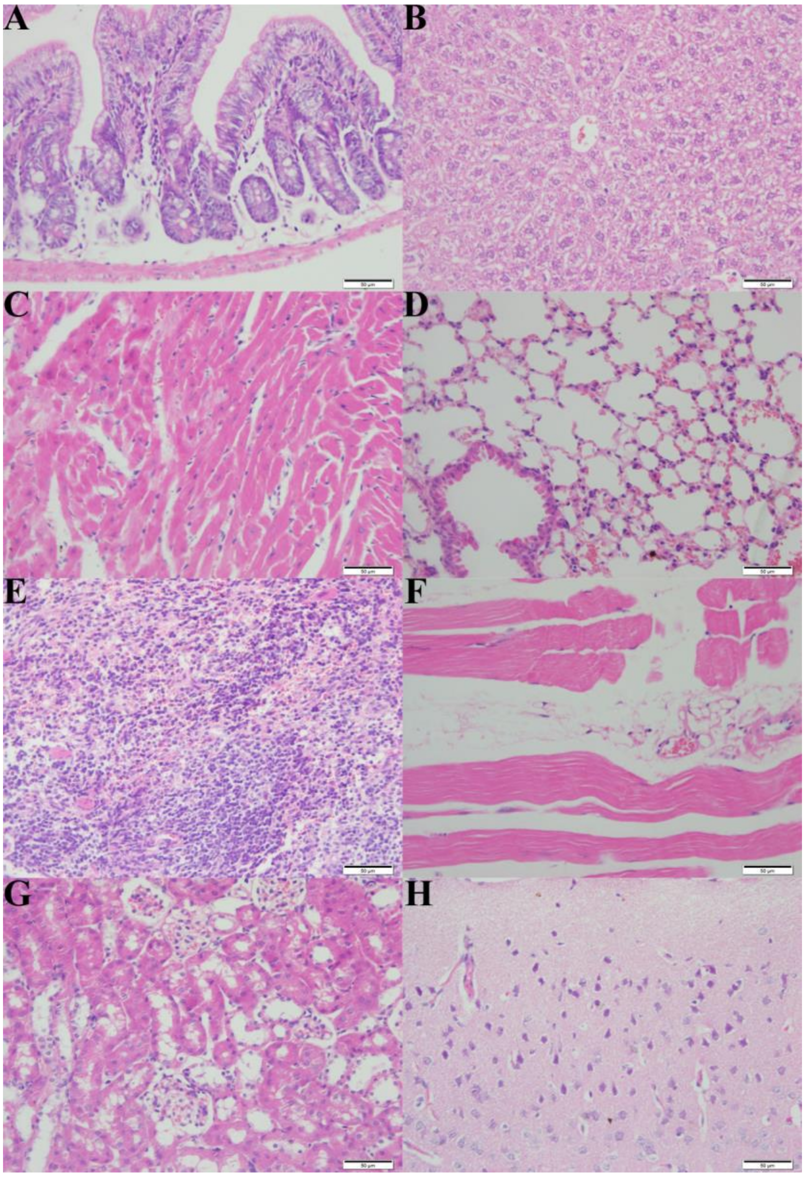

Supplementary figure 1 Morphology of tissues of transgenic RNAi mouse. A: intestine, B: liver, C: heart, D: lung, E: spleen, F: muscle, G: kidney, H: brain

Scale bar, 50 μm for 40×.
